# An Acoustic Emission Method for Assessing the Degree of Degradation of Mechanical Properties and Residual Life of Metal Structures under Complex Dynamic Deformation Stresses

**DOI:** 10.3390/ma14092090

**Published:** 2021-04-21

**Authors:** Petr Louda, Artem Sharko, Dmitry Stepanchikov

**Affiliations:** 1 Department of Material Science, Technical University of Liberec, 461 17 Liberec, Czech Republic; petr.louda@tul.cz; 2 Department of Automation and Computer-Integrated Technologies, Kherson National Technical University, 73008 Kherson, Ukraine; 3 Department of Energetics, Electrical Engineering and Physics, Kherson National Technical University, 73008 Kherson, Ukraine; dmitro_step75@ukr.net

**Keywords:** identification, deformation, condition, mechanical properties, residual life, complex loads, acoustic emission

## Abstract

An acoustic emission method for assessing the degree of degradation of mechanical properties under conditions of complex dynamic deformation stresses is proposed. It has been shown that changing the operating conditions of metal structures, peak loads, external collisions, and thermally changing loads, which cannot be taken into account, leads to uncertainty and unpredictable structural changes in the material. This in turn makes it difficult to identify the state of the structure material to ensure trouble-free operation of the equipment. Changes in the mechanical properties under difficult loading conditions are identified by polynomial approximation of the results of AE measurements and the construction of boundary curves separating the operability region from the fracture region. This is achieved by approximating the experimental dependences of the acoustic parameters for various types of loading. This approach significantly expands the capabilities of the technical means of identification systems of metal structures, and in particular, allows the current state of the equipment and its suitability for further operation to be assessed without stopping the equipment in real time. It is of interest not only to fix the damage, but also to diagnose the processes of reducing the mechanical properties during the operation of the equipment.

## 1. Introduction

Determination of the degree of degradation of mechanical properties and the residual life of metal structures under complex dynamic deformation stresses has various applications related to bending, tensile static and dynamic loads, and processes of the initiation of defects.

The trouble-free operation of metal structures relies on the monitoring of their technical condition and the implementation of physical diagnostic methods. The need to conduct an industrial safety examination exists for equipment that has fully run its service life or has been used under conditions not prescribed by the standards, when emergency situations may have occurred, which may affect the performance of the material.

To assess the state of materials, it is sufficient not only to determine the standard mechanical properties, but also the properties that are sensitive to local structural changes manifested in microplastic deformation and the occurrence of micro-stresses. Accumulation of damage and degradation of the material structure under complex dynamic deformation stresses causes structural degradation processes of deformation and destruction of cementite in pearlite columns, and dislocation substructure evolution. This leads to a significant reduction in brittle fracture resistance characteristics.

The operating time of an object from the beginning of its operation to the onset of the limiting state is considered a resource. When assessing the operability of structures associated with types of loading, the safety factor is understood as the ratio of the residual life to the current operating time. Degradation of the material during the operation of structures causes a decrease in their resource. Residual life is the difference between the limit and current state of the material during operation.

Further resource assessments include an assessment of the current state, prediction of this condition under conditions of normal equipment operation, an assessment of the probability of failures, and an assessment of the risks of emergency situations. Analysis of synthetic accidents at hazardous production facilities consists of the systematic use of all the available information to identify hazards and undesirable events. To assess this, statistical data on the accident rate and reliability of technological systems, logical and simulation methods are used, e.g., the tree of events and failures, expert methods, and assessment of consequences.

The main analyzed characteristics include safety margins according to the criteria of static strength, low and high-cycle fatigue, and initial and residual life.

The efficiency of metal structures is determined by their strength and permanent deformations through displacements, deflections and turns. The resource of long-term loaded structures is determined by the processes occurring under conditions of elastic deformation. Deformations may be localized near the place of collision of the contacting parts of metal structures, but they may also be general, involving the entire volume of the collision. In practice, both types of deformation occur simultaneously.

The key role in identifying the state of a metal structure is played by resistance to deformation, which is determined both under uniaxial tension and compression of samples.

Determination of the degree of critical damageability of the material structure is of particular scientific and practical interest.

The aim of this work is to use the phenomenon of acoustic emission to address the issue of predicting changes in the mechanical properties of materials under loading.

## 2. Relative Works

The study of metal under load requires the use of disciplines such as materials science, mechanical testing, and technical diagnostics.

During the operation of structures, periodic control of deformation deviations from the design values and static monitoring of deformations are performed [1,2,3,4,5,6]. There are several traditional methods for assessing stresses, e.g., the method of drilling holes, the method for determining stresses by measuring the surface hardness [7], methods using surface waves [8,9], the phenomenon of acoustoelasticity and interferometry [10,11], X-ray methods [12], electromagnetic methods [13], tensometric methods [14], magnetic resonance methods [15], and acoustic emission [16].

Modern diagnostic methods assessing the degree of degradation of the mechanical properties of materials have various technical applications [17,18]. In [19], the identification and forecasting of the turning points of bifurcations using complex networks is presented. In [20], the results of the identification of complex interactions between biological objects and their modeling in the form of graphs are presented. In [21], the results of the identification of hazardous characteristics of a thermal runaway of powder ejected from lithium-ion batteries are presented. An algorithm for identifying unknown parameters of photovoltaic systems is described in [22]. A modified algorithm for identifying structural damage at varying temperature and its effect on Young’s modulus is presented in [23]. New materials for measuring forces and deformations in a wide range of loads of external factors and radiation resistance are presented in [24].

The joint use of the strain gauge method with the finite element method for assessing deformation is presented in [25]. The development of methods for determining the strain-stress state of a load-bearing structure by determining deformations at tensometric points is presented in [26].

The identification of the critical states of serviceability and transition boundaries of the stability of power systems with multidimensional uncertainties when replacing the state space with a parametric space is presented in [27]. In [28], a method is presented for calculating the parameters of mechanical tensile strength, based on measurements of deformation using strain gauges with subsequent modeling.

In [29], the results of assessing the accumulated damage based on the determination of the values of the diagnostic parameter of acoustic emission are presented. The possibilities and prospects of acoustic emission diagnostics in assessing the structural state of metallic materials at various stages of loading are reflected in [30,31,32,33].

In [34], an algorithm is described, which connects such parameters of the state of an object as residual life, the moment of onset of plastic deformation with the parameters of acoustic emission diagnostics. The object of the study was a polymer material brought to failure with a tensile load. Recently, there has been a constant interest in the study of the properties of new polymer materials by physical test methods [35,36].

An analysis of the existing methods and means for determining the degradation of the mechanical properties of materials during the operation of structures in real time showed that they are all intended for the study of one type of deformation: tension [37,38,39] or bending [40,41,42,43], while under real conditions loading forces act on the material, under combined conditions of deformation [44,45,46,47,48,49,50].

## 3. Materials and Methods

The operability states of equipment during its operation are identified by means of technical diagnostics and non-destructive testing. The quality indicators of metal structures change when interacting with the environment and other organizational and technical objects. The loading parameter is permanent deformation. To identify the state of metal structures under conditions of complex deformation stresses on the material, strength calculations should consider the kinetics of destruction, characterized by the rate of damage accumulation, such as creep and cyclic fatigue.

The emergence of elastic and plastic deformations in materials is inevitably accompanied by the appearance of internal and external stresses. Internal stresses arise due to the inhomogeneous flow of structural and phase transformations in metals, in the processes of rapid heating or cooling of the metal, due to inhomogeneous deformation of the surface and inner layers. External stresses are dependent on operating conditions. There is a balance between internal elastic forces and external stresses. With an increase in the external operating load, the internal elastic forces also increase.

Existing methods of strength verification calculations include calculations for the corrosion rate of metal, calculations for crack resistance, calculations for metal fatigue, and creep calculations. These techniques imply independent processes of corrosion, fatigue and creep, although in practice these processes occur simultaneously and in different combinations.

The disadvantages of these methods include the uncertainty of estimates, low accuracy of results, and the need to analyze the compliance of operating conditions with industrial safety requirements.

For a theoretical description of the degradation of mechanical properties, the most suitable method is a mathematical model of the energy spectrum of AE signals. The energy spectrum of acoustic emission signals from nanoscale objects in the form of point masses *m*, connected by elastic bonds is presented in [51].

The potential energy of such a chain is

(1) $$\Phi =\Phi_{0}+\sum_{n}\Phi \left(n\right)x\left(n\right)+\frac{1}{2}\sum_{n,n_{1}}\Phi \left(n,n_{1}\right)x\left(n\right)x\left(n_{1}\right)+\frac{1}{3!}\sum_{n,n_{1},n_{2}}\Phi \left(n,n_{1},n_{2}\right)x\left(n_{1}\right)x\left(n_{2}\right)+\ldots ,$$

where Φ_0_–energy of a linear chain in equilibrium, *n*, *n*_1_, *n*_2_–numbers of interacting particles *x*(*n*)—displacement.

The kinetic energy of such a chain is

(2) $$T=\frac{m}{2}\sum_{n}{\left[\frac{\partial x\left(n,t\right)}{\partial t}\right]}^{2}$$

The common difference between the kinetic and potential energy determines the Lagrange function.

(3) $$L=\frac{m}{2}\sum_{n}{\left[\frac{\partial x\left(n,t\right)}{\partial t}\right]}^{2}-\frac{1}{2}\sum_{n,n_{1}}\Phi \left(n,n_{1}\right)x\left(n,t\right)x\left(n_{1},t\right)+\sum_{n}F\left(n,t\right)x\left(n,t\right)$$

where *F*(*n*,*t*)—external force.

The spectrum of the acoustic emission signal may be represented not only on the time *t*, but also on the frequency *ω*. For this, the Fourier transform *x*(*ω*) is used

(4) $$x\left(\omega \right)=\int x\left(t\right)e^{i\omega t}dt;\;x\left(t\right)=\frac{1}{2\pi }\int x\left(\omega \right)e^{-i\omega t}d\omega$$

The oscillatory properties of a discrete microstructure under load are taken into account in the model of a diatomic cell [52].

The translational and rotational properties in such a cell are expressed in terms of displacement of particles in the middle of the cell *y*(*n*) and center of mass displacement *x*(*n*)

(5) $$x(n)=\frac{1}{m}\left[m_{1}\omega (n,1)+m_{2}\omega (n,2)\right]$$

(6) $$y(n)=\frac{m_{1}\xi_{1}\omega (n,1)+m_{2}\xi_{2}\omega (n,2)}{I}$$

where *I*–cell moment of inertia, *ξ*_1_ and *ξ*_2_–particle coordinates relative to the center of mass, *m* = *m*_1_ + *m*_2_*, I* = *m*_1_*ξ*_1_^2^ + *m*_2_*ξ*_2_^2^.

By denoting the summarized forces corresponding to *x*(*n*) and *y*(*n*) by *F_x_*(*n*) and *F_y_*(*n*), we obtain the equation of motion of the entire cell in the medium:

(7) $$m\frac{\partial^{2}x\left(n,t\right)}{\partial t^{2}}x\left(n\right)+\sum_{n}\Phi^{00}\left(n-n_{1}\right)x\left(n_{1}\right)+\sum_{n_{1}}\Phi^{01}\left(n-n_{1}\right)y\left(n_{1}\right)=F_{x}\left(n\right)$$

(8) $$I\frac{\partial y\left(n\right)}{\partial t}x\left(n\right)+\sum_{n_{1}}\Phi^{10}\left(n-n_{1}\right)x\left(n_{1}\right)+\sum_{n_{1}}\Phi^{11}\left(n-n_{1}\right)y\left(n_{1}\right)=F_{y}\left(n\right)$$

In this case, *x*(*n*) determines the high-frequency, and *y*(*n*)-the low-frequency part of the acoustic emission spectrum.

This provides a connection between the translational and rotational degrees of freedom and the oscillatory properties of particles.

In the model of a continuous medium in the form of a linear chain, atoms interact through central forces. This model is well suited for describing densely packed structures. In the moment model, the interaction between atoms remains paired, but is carried out using the moments.

Since the numbers of interacting particles are not uniquely determined, then for Φ (*n, n*_1_) it is necessary to fulfill the conditions of mirror symmetry:

(9) $$\Phi \left(n,n_{1}\right)=\Phi \left(n_{1},n\right)$$

For the numbering of particles in a diatomic cell, the Weyl symmetry is used, in which a transformation that preserves the structure of the space is an automorphism.

The notion of automorphisms is used here as Fourier transforms. Automorphisms translates an acoustic signal caused by a change in the structure of a material into a function of trigonometric sums

(10) $$\sum_{n}e^{2\pi if(n)}=\sum_{n}\cos[2\pi f(n)]+\sum_{n}\sin[2\pi f(n)]$$

where *f*(*n*)—a polynomial with rational coefficients.

The change in the state of the medium is expressed in the form of the differential equation of the precursor of destruction **X** *(t)* [53].

(11) $$\frac{d}{dt}X(t)=A(t)X(t)+B(t)C(t),X(t_{0})=X_{0}$$

where **A** (*t*) is a matrix of the characteristics of AE signals, **B** (*t*) is a matrix of sensitivity to noise, **C** (*t*) is a column vector of the low-frequency component caused by hardware inaccuracies.

The system of equations for the initiation of changes in the structure of materials for a model of a complex structure under equilibrium conditions has the following form:

(12) $$a\sum_{n}n^{2}\Phi^{00}(n)-2\sum_{n}n\Phi^{01}(n)=0$$

(13) $$a\sum_{n}n\Phi^{10}(n)-\sum_{n}n\Phi^{11}(n)=0$$

where *a*—distance between centers of mass in a diatomic cell.

The matrix Φ*^ik^*(*n*) (*i*, *k* = 0, 1) is expressed coordinate-wise through the force constants Φ*^ik^*(*n*).

The methodology for determining the residual resource consists of the implementation of the provisions that discrete structure of nanoscale objects is characterized by a transition from a system of linear equations to differential equations of oscillating waves behind the destruction front.

St3sp grade structural carbon steel was chosen as a material for the research. This steel is widely used in industry, mechanical engineering and construction for the manufacture of load-bearing elements of welded and complex structures operating at normal and positive temperatures (Table 1).

The widespread use of this group of materials is possible by a whole set of physical, mechanical and technological characteristics that ensure effective and long-term operation of products. Diagnostics of technical structures made of carbon steels is an urgent task of modern technology.

Motivation for the significance of our research requires the involvement of additional information about the selected material.

The physical and mechanical properties of this group of materials are determined in accordance with the tensile diagram. At the initial stage of loading, the tensile diagram is a straight line expressing the proportionality between load and deformation. The corresponding zone is the elastic deformation zone, which ends with the value of the proportional limit *σ_pr_*.

(14) $$\sigma_{pr}=\frac{P_{pr}}{S_{0}}$$

where *Ppr* is the load at which the proportionality of loading and deformation is not violated, *S*_0_ is the initial cross-sectional area of the specimen.

For St3sp grade steel there is a fairly wide scatter of this value, determined from the results of mechanical tests *σ_pr_* = 195–200 MPa. After reaching *σ_pr_*, the deformation begins to grow faster than the load and the diagram becomes curvilinear. The elastic limit characterizes the transition from elastic to plastic deformation zones. For St3sp grade steel, the elastic limit is 205–210 MPa. In this region, deformations grow without increasing the load. This region is the yield zone or pre-fracture.

(15) $$\sigma_{yi}=\sigma_{0.2}=\frac{P_{yi}}{S_{0}}—\mathrm{yield}\;\mathrm{point}$$

where *P_yi_* is the load at which the specimen deforms without increasing the load.

For structural low-carbon steels, the tensile diagram of which does not have a pronounced yield area, the yield stress is conventionally defined as the stress at which the residual deformation is 0.2% of the calculated length of the sample. This value is denoted as *σ*_0.2_. For St3sp grade steel *σ*_0.2_ = 210–255 MPa.

The stress corresponding to the highest load preceding the failure of the specimen is called the breaking point.

(16) $$\sigma_{B}=\frac{P_{\max}}{S_{0}}—\mathrm{breaking}\;\mathrm{point}$$

where *P*_max_ is the maximum ultimate load, after which the specimen begins to constrict in the form of a neck.

For St3sp grade steel *σ*_B_ = 370–490 MPa.

The yield zone is followed by a zone of breaking or pre-fracture. Here, the elongation of the specimen occurs simultaneously along the entire length. At maximum effort, a neck appears, and further deformation occurs in this zone. The section in the middle of the neck decreases rapidly, and the stresses in this place rapidly increase, although the tensile forces decrease. Outside the neck region, the stresses decrease, and the rest of the sample does not elongate. Then, the sample is destroyed.

(17) $$\sigma_{d}=\frac{P_{d}}{S_{0}}—\mathrm{destroying}\;\mathrm{stress}$$

where *P_d_* is the load at which the sample is destroyed.

During tension, the cross section of the specimen changes continuously, especially when loaded above the yield point; therefore, the values of *σ*_B_ and *σ_d_* are rather conventional. Starting from the load *P*_max_, the specimen shrinks and necks. At the moment of sample rupture, the neck section turns out to be less than the initial sample section area.

The disadvantage of determining the physical and mechanical properties from the tensile diagram is the low sensitivity of mechanical tests to changes in the load caused by a change in the structure of materials.

Acoustic emission measurements of tensile and bending diagrams are free from these disadvantages. This makes it possible to record the onset of characteristic zones of work hardening earlier than it follows from the results of mechanical tests. This ensures the accuracy and adequacy of their estimates.

Acoustic measurements of the state of the material under load allow the use of only one type of sensor. This approach greatly simplifies the procedure for assessing the residual life of metal structures in continuous operation, and allows the calculation process to be automated.

## 4. Experiment

The dynamics of physical processes accompanying AE radiation are described by the amplitude-time distribution *n*(*A,t*)*,* i.e., a function indicating the number of AE pulses *dN_A,t_* recorded in the time interval from *t* to *t + dt*, with an amplitude in the interval from *A* to *A + dA*:

(18) $$dN_{A,t}=n(A,t)dAdt$$

If the total time of AE registration is in the range from 0 to *T*, then:

(19) $$N_{A,t}=\int_{0}^{T}\int_{A_{\min}}^{A_{\max}}n(A,t)dAdt$$

As an informative parameter of the AE signal was determined by counting the number of crossings of the signal threshold level divided by the signal observation time. By analogy with physical quantities, this parameter is called the density of the AE signal. The threshold level was taken equal to *A*_min_ = 0.05*U*_max_ for each signal. In contrast to such generally accepted acoustic emission parameters as the number of AE acts, the cumulative amount, it is more convenient parameter to using in measurement process [54].

As a result of the experiments, it was found that the qualitative informative characteristic of the AE signals of the samples under load is the amplitude distribution density in time:

(20) $$N=\frac{N_{A,t}}{T}$$

To determine the safety factor under multiparameter loading, one should consider how the deformations change when the loading parameters change with respect to the limiting condition.

In [55], the strength of rocket structures is defined as the correspondence of the development trajectory of the material degradation process to the boundary curve constructed during mechanical bending and tensile tests. To carry out such a control using the acoustic emission method, it is necessary to know the correlations between the mechanical and deformation characteristics of metals and the AE parameters under various test conditions.

The results of acoustic emission measurements performed on St3sp grade steel are included in [56,57].

Serviceability is determined based on industrial safety standards and regulations, practical experience in operating a hazardous production facility, a list of undesirable events, and a description of hazard sources.

The general trend in the assessment of the residual life is the transition from failure statistics to an integrated approach that combines the results of destructive and non-destructive testing with strength verification calculations.

The optimization criterion is the correlation coefficient of the values of the diagnostic parameter and the indicator of the operability of the metal structure.

In practice, the area of operability of metal structures is limited by Hooke’s law, where deformations are proportional to the applied stress. However, when operating metal structures under uncertainty conditions, stresses may exceed the area limited by Hooke’s law. Therefore, functional relations for tension *σ_t_* = *f*(*ε_t_*) and for bend *σ_b_* = *f*(*ε_b_*) must correctly describe the entire loading diagram, starting from the proportional range and ending with the ultimate strength.

**Figure 1:**
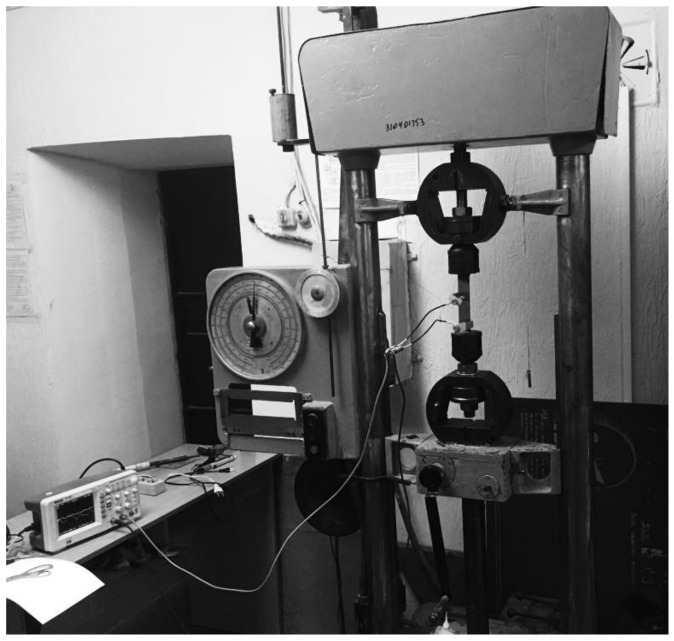
The experimental device for the synchronous measurement of AE signals with uniaxial loading tests.

A device for the synchronous measurement of AE signals with uniaxial loading tests is shown in Figure 1.

The measuring setup used broadband acoustic sensors for the AF15 acoustic emission device. Signals after amplification were recorded with a RIGOL DS1052E digital oscilloscope. In this case, the signal was recorded in a digital format.

Measurement of the deformation of the samples under uniaxial loading was determined by fixing the elongation using a Micron digital indicator such as a DT-7011 micrometric electronic displacement indicator. The micrometric electronic indicator of displacement, in contrast to the mechanical one, is adjusted to zero at any point of the scale, so that it is possible to observe the deviation from the dimensions of the test sample in the range of 0–12.7 mm with an inaccuracy of 0.001 mm.

The micrometric electronic indicator of movement is based on a capacitive matrix-encoder, which consists of two series-connected capacitors that form a digital vernier.

During operation, the device with one end through a clamp was attached to the upper grip of the UM5 tensile testing machine, touching the indicator stand, which is a micrometric screw located parallel to the test sample. The other end of the indicator stand was attached through a clamp to the lower grip of the tensile testing machine.

Non-informative acoustic emission signals caused by noise and friction in the gripping area of the tensile testing machine were reduced by using rubber gaskets between the sensors and the sample in the sensor clamp.

**Figure 2:**
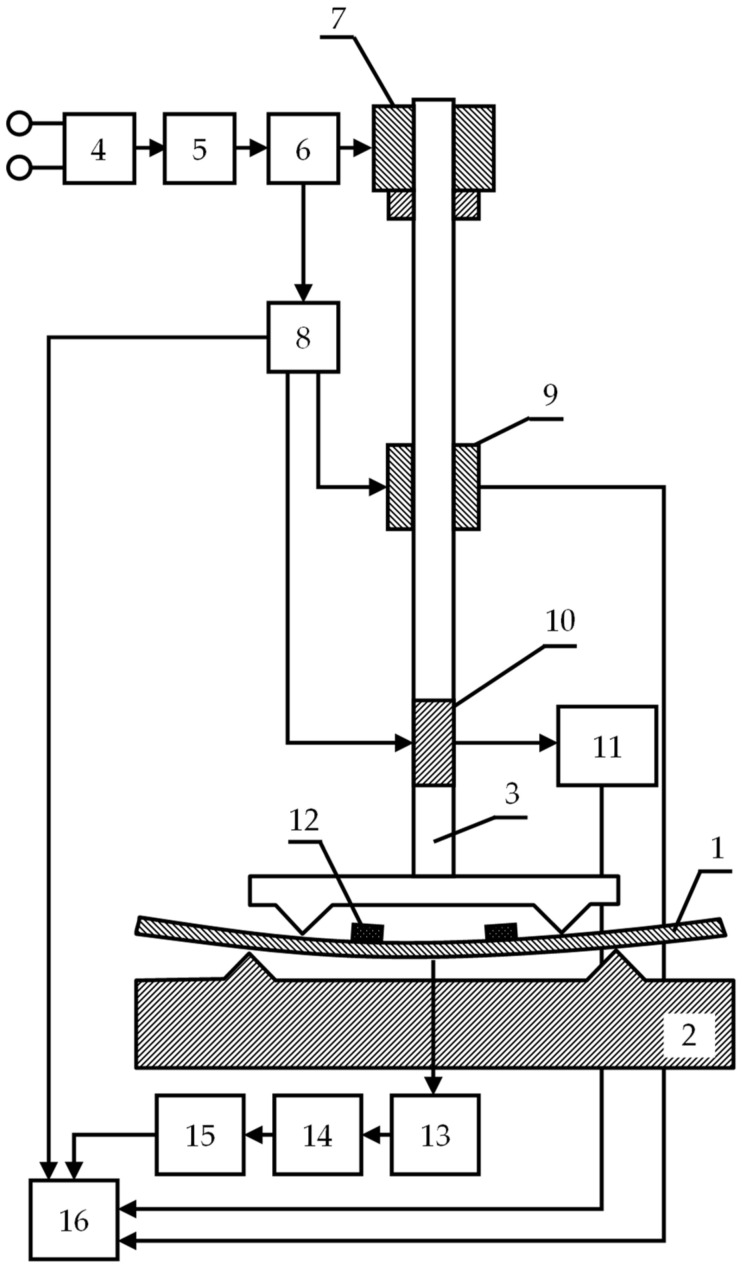
The block scheme of AE device for fixing bending deformations.

A block diagram of the experimental setup for four-point bending tests is shown in Figure 2.

During testing, the sample (1) is installed between the prisms of the MIP-10 testing machine (2), designed for testing springs and modernized for testing for four-point bending, and an identifier in the form of a loading device rod (3). The loading device (4) consists of a pulsed DC power supply unit (5), a drive unit (6) and a planetary gearbox (7). A counter for counting the time from the start of loading (8) is installed on the drive unit (6). An acoustic emission sensor (12) is installed directly on the sample at its end. To assess the degree of loading of the sample, simultaneously with the timer (8), the signal is fed to the strain gauge (9) and to the digital deflection indicator (10) and then through the microprocessor (11) to the computer (16). A signal from the AE sensor (12) is also fed here through the information unit (13), the unit for accumulating and processing information (14), and the AE recording device (15). The recording device may be made in the form of a storage oscilloscope. Therefore, the computer simultaneously collects information from the parameters of the loading device.

During the tests, information on the values of deformation and stresses at the current time, together with the AE information, is written into a file with a specified frequency.

**Figure 3:**
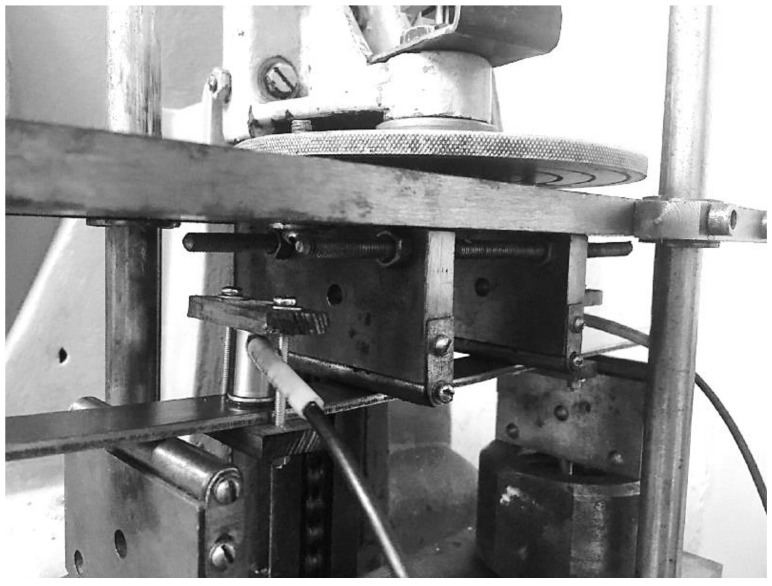
The acoustic emission device for studying bending deformation.

An acoustic emission device for studying bending deformation is shown in Figure 3.

In terms of construction, the four-point bending test device contains two main mechanisms: deformation and fixing power load. The deformation mechanism includes: a motor, a worm gear, a loading screw, a carriage with an upper plate, and a screw for disconnecting the compensation spring. The force measuring mechanism includes: a lower plate and a lever-type transmission mechanism located inside the housing. The installation also contains punches in the form of a loading device indenter and a support. The support was fixed in place to allow precise centering of the sample. In this case, the longitudinal axis of the sample was located parallel to the lateral surface of the traverse, and the center of symmetry of the sample coincided with the axis of the applied load. The AE sensors were attached to the sample with the help of clamps. During the tests, the principle of synchronization with registration of load, deformation and AE measurements was implemented.

The measuring setups used broadband sensors for an AF15 acoustic emission device with a bandwidth of 0.2–2.0 MHz. In this case, frequency filtering was performed. Limitations in the low-frequency range are caused by the need to cut off the noise of the mechanical test equipment, and in the high-frequency range, by the need to cut off electromagnetic interference.

During the tests, the AE signals from two sensors were analyzed, the placement of which on the sample is shown in Figure 2, pos. 12. Samples for the uniaxial tensile tests were cut from a rolled sheet with the dimensions of 223 × 37 × 3 mm, and 300 × 20 × 4 mm for the four-point bending tests. For each type of test, five specimens were used.

The dynamic characteristic of the acoustic emission signals is the intensity of AE, which is determined by dividing the total number of pulses *N_A,t_* by the observed time interval, which for the RIGOL DS1052E digital oscilloscope was 2 μs.

The chemical composition of St3sp grade steel is shown in Table 2.

## 5. Results and Discussion

The strength properties of the material are characterized by the ultimate strength *σ*_B_, yield point *σ*_0.2_, and deformation properties-relative deformation of fracture in tension *ε*_B_ and bend *ε*_0.2_. For test specimens made of St3sp grade steel, these parameters are equal to *σ*_B_ = 490.3 MPa, σ_0.2_ = 240 MPa, *ε*_B_ = 7.80%, *ε*_0.2_ = 4.19%. The conditions for the performance of the material will be the fulfillment of the conditions *σ_t_* < *σ*_B_ or ε*_t_* < ε_B_ for tensile deformation and *σ_b_* < *σ*_0.2_ or *ε_b_* < *ε*_0.2_ for bending deformation.

**Figure 4:**
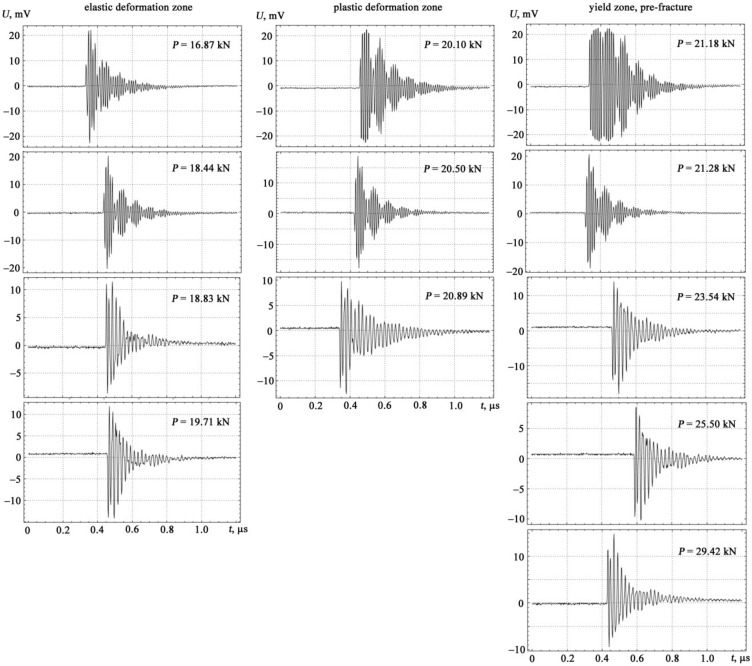
Experimental investigation of the form of acoustic emission signals under tension loading.

**Figure 5:**
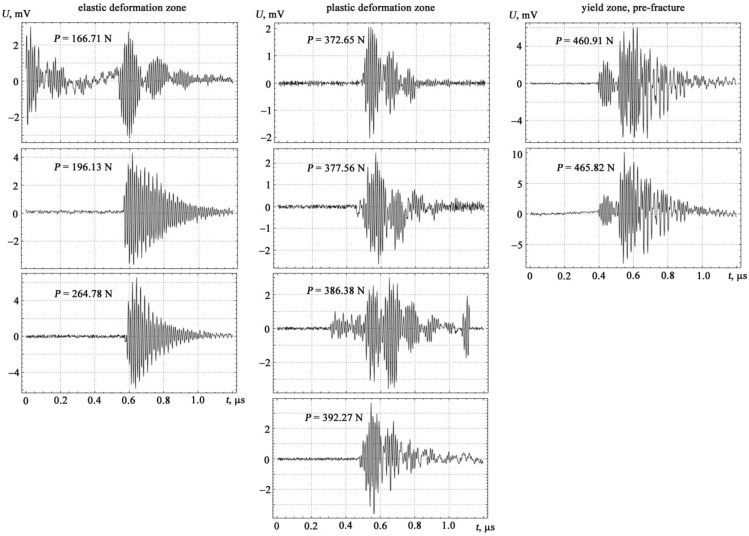
Experimental investigation of the form of acoustic emission signals under transverse bending loads.

If the spectra of acoustic emission signals the tension and the bending are plotted, taking the tension diagram as a basis, then they form three characteristic zones: elastic deformation, plastic deformation and yield zone, pre-facture up to the destruction of the specimen with increasing load (see Figure 4 and Figure 5).

**Figure 6:**
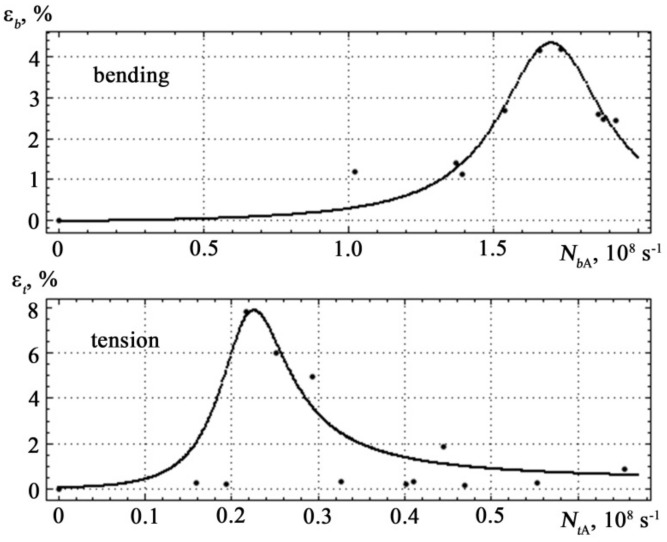
Dependences of the AE signal density on the relative deformation of the St3sp grade steel specimen. The dots show the experimental values, the solid line is the approximation.

To use the obtained experimental data to determine the degradation of the mechanical properties of materials under difficult loading conditions, the correspondence of the relative deformation on the density *N* of the AE signals was approximated *ε_t_*(*N*) for tensile and bending *ε_b_*(*N*). When choosing a mathematical function for the approximation, the determining criteria were the maximum possible coefficient of determination (close to 1) and the minimum root-mean-square inaccuracy. The form of the mathematical function that meets these criteria is shown in Figure 6 and is described by equations.

(21) $$\varepsilon_{t}\left(N\right)=\frac{-0.094N^{2}+0.348N-0.108}{N^{2}-3.394N+2.929}$$

(22) $$\varepsilon_{b}\left(N\right)=\frac{0.238N^{2}+0.022N+0.002}{N^{2}-0.436N+0.049}$$

The different scale of the curve in Figure 6 is explained by the fact that the density of the AE signal during tensile deformation differs by an order of magnitude from the signal density during bending deformation. This feature allows clear definition of the type of mechanical stress in the specimen *σ_t_ σ_b_*. Analysis of Figure 6 shows that the density of the AE signal during bending *N_bA_* is an order of magnitude higher than the density of the AE signal *N_tA_* during tension. When diagnosing a specimen with a complex nature of loading (longitudinal tension and transverse bending), this property will make it possible to clearly determine the type of mechanical stress in the specimen-tension and bending, respectively.

**Figure 7:**
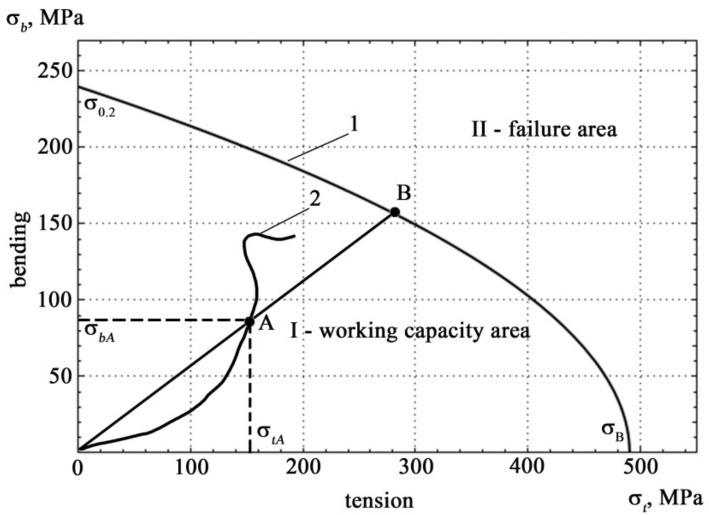
Boundary curve 1 and possible loading trajectory 2 in the coordinates of mechanical stress.

The boundary curve and possible loading trajectory in the coordinates of mechanical stress are shown in Figure 7.

A technology for identifying the state of metal structures is proposed, which is distinguished by the construction of the boundary surface of destruction using acoustic measurements. This requires the construction of the fracture boundary surface in the coordinates of the density of acoustic signals under combined tensile and bending deformation. The boundary curve is drawn from separate measurements of tension and bending.

Based on the experimental data in [56,57], it is possible to obtain the correspondences of the mechanical stress on deformation in tension *σ_t_*(*ε_t_*) and bending *σ_b_*(*ε_b_*), which describe all sections of the loading diagram:

(23) $$\sigma_{t}\left(\varepsilon_{t}\right)=337.7\exp\left(0.042\varepsilon_{t}\right)-338.4\exp\left(-11.710\varepsilon_{t}\right)$$

(24) $$\sigma_{b}\left(\varepsilon_{b}\right)=\frac{9.723\times 10^{6}\varepsilon_{b}-9.451\times 10^{4}}{\varepsilon_{b}^{3}+8803\varepsilon_{b}^{2}-2.343\times 10^{4}\varepsilon_{b}^{}+1.171\times 10^{5}}$$

**Figure 8:**
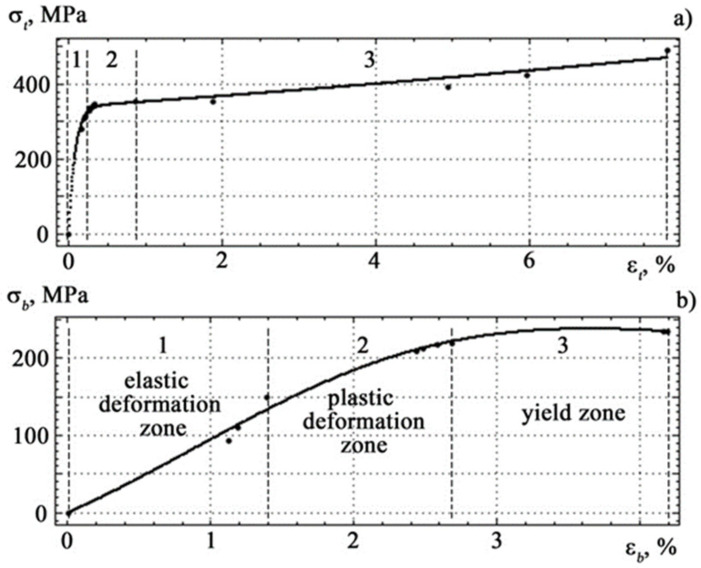
Relationship between strength and deformation properties under different loading conditions for tension (**a**) and bending (**b**).

The graphical form of Equations (23) and (24) is shown in Figure 8. 

**Figure 9:**
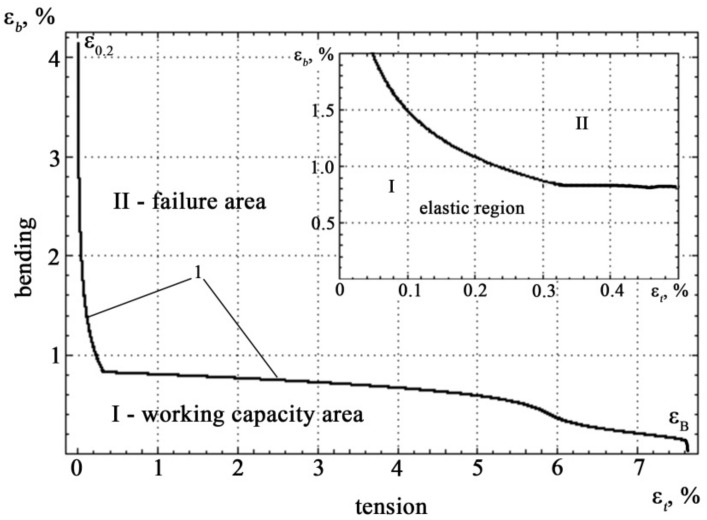
Boundary curve 1 and areas of working capacity and fracture states in the coordinates of relative deformation (the inset shows a portion of the boundary curve corresponding to the elastic deformation zone).

The resulting correspondence allow the boundary curve to be rebuilt in the coordinates of the relative deformation. The results of this construction are shown in Figure 9.

Since the normal operation of metal structures should take place in the area of loading and elastic deformations corresponding to Hooke’s law, it has a special practical significance for it, similar to the areas of state and destruction.

Analysis of Figure 9 shows that on the boundary curve there are two clearly defined sections with different nature of the correspondence *ε_b_* = *f*(*ε_t_*). 

**Figure 10:**
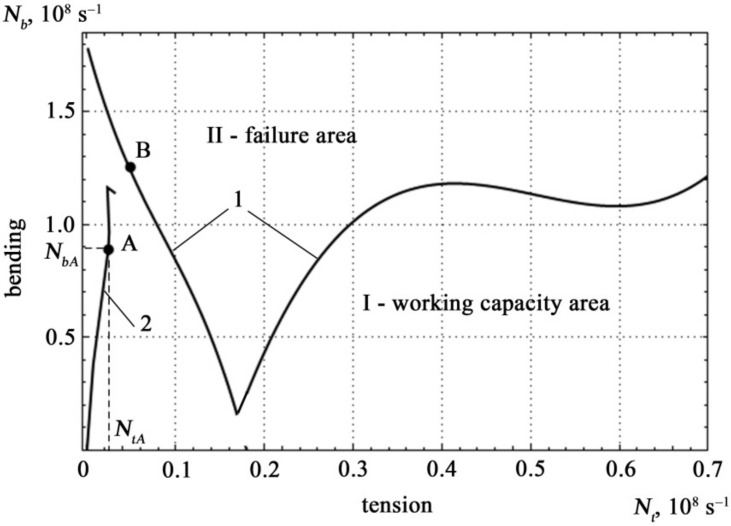
Boundary curve 1, possible loading trajectory 2 and areas of working capacity and fracture states under tensile and bending deformations.

The values of working capacity and fracture states determined from the results of measurements of the density of acoustic emission signals are shown in Figure 10.

**Figure 11:**
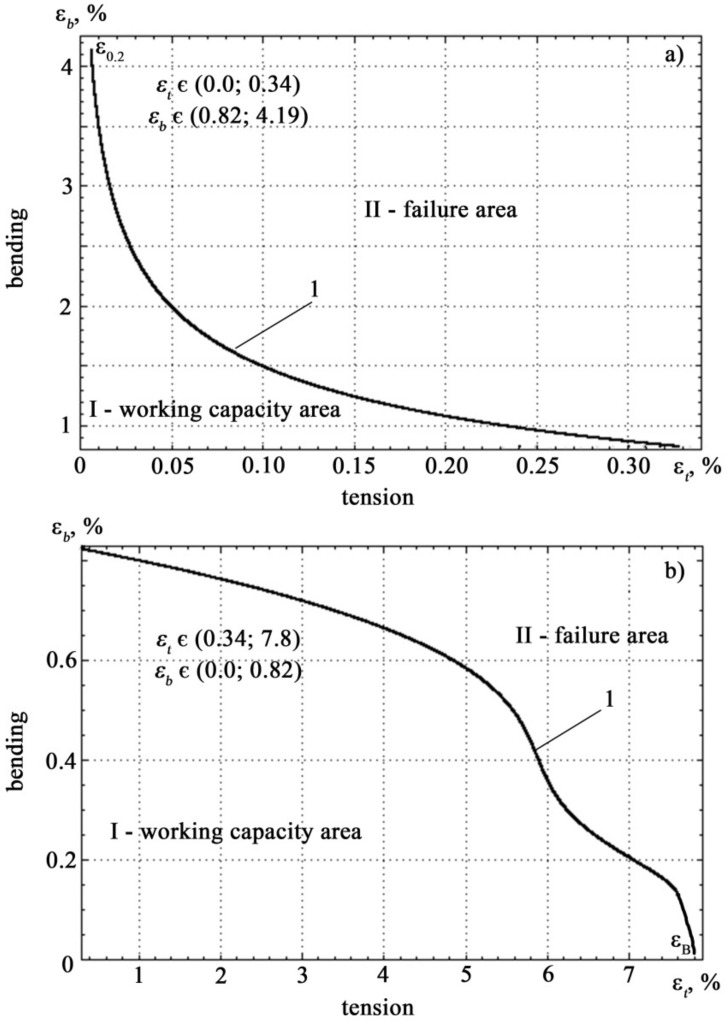
Sections of boundary curve 1 corresponding to the different nature of the *ε_b_*(*ε_t_*) dependence: section (**a**) is characterized by a slow change in tensile strain with large changes in bending strain, section (**b**) is characterized by a rapid change in tensile strain with insignificant changes in bending strain.

Figure 11 shows a region of the boundary curve in the coordinates of relative deformation. In Figure 11a the section is characterized by a rapid change in tensile strain with insignificant changes in bending strain and corresponds to the values of the relative tensile strain in the range *ε_t_* ∈ (0, 0.34) and bending in the range *ε_b_* ∈ (0.82, 4.19), the section on Figure 11b is characterized by a slow change in tensile strain with large changes in bending strain and corresponds to the values of the relative tensile strain in the range *ε_t_* ∈ (0.34, 7.8) and bending in the range *ε_b_* ∈ (0, 0.82).

It is clear that the limiting curve has two regions: the first region in the interval *N_t_*∈[0, 0.17], the second region in the interval *N_t_*∈[0.17, 0.7]. Polynomial approximations of the boundary curve are presented in the form of equations

(25) $$N_{b}\left(N_{t}\right)=-306.60N_{t}^{3}+80.50N_{t}^{2}-14.53N_{t}+1.79\;;\;N_{t}\in \left[0,\;0.17\right]$$

(26) $$N_{b}\left(N_{t}\right)=33.43N_{t}^{3}-50.68N_{t}^{2}+24.78N_{t}-2.76\;;\;N_{t}\in \left[0.17,\;0.7\right]$$

The technology for determining the strength and deformation characteristics of metal structures using the acoustic emission method in real time is as follows. The values of the corresponding tensile and bending deformations are calculated for specific measured values of the density of acoustic signals, using the formulas of the approximating dependences of the deformation characteristics on the parameters of the AE signals. The strength characteristics of the material at a given time are calculated by empirical dependences and their approximating functions.

The geometric interpretation of the safety factor *λ* at point A in Figure 7 is represented as Equation (27):

(27) $$\lambda =\frac{OB}{OA}\geq 1$$

The analytical safety factor *λ* for point A on curve 2 in Figure 7 is described by formula [58]:

(28) $$\lambda =\frac{\sigma_{0.2}}{\sigma_{bA}^{}}\left[-\frac{1}{2}\frac{\sigma_{0.2}\sigma_{tA}\left(\varepsilon_{t}\right)}{\sigma_{B}\sigma_{bA}\left(\varepsilon_{b}\right)}+\sqrt{\frac{1}{4}{\left(\frac{\sigma_{0.2}\sigma_{tA}\left(\varepsilon_{t}\right)}{\sigma_{B}\sigma_{bA}\left(\varepsilon_{b}\right)}\right)}^{2}+1}\right]$$

**Figure 12:**
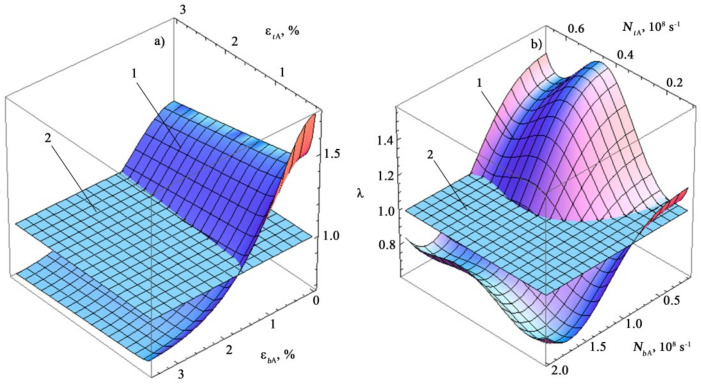
Three-dimensional image of the safety factor depending on changes in acoustic (**a**) and deformation (**b**) characteristics under different types of loading: 1-response surface, 2-equilibrium surface λ = 1.

Graphical representations of the safety factor *λ* as the surfaces $\lambda =f\left(\varepsilon_{tA},\varepsilon_{bA}\right)$ and $\lambda =f\left(N_{tA},N_{bA}\right)$ are shown in Figure 12.

In Figure 12, the element of the response surface located above the equilibrium surface characterizes the destruction region. The intersection line of these surfaces is the boundary curve (see Figure 9, Figure 10 and Figure 11).

Therefore, the obtained Equation (28) makes it possible to estimate the safety factor of a metal sample made of St3sp grade steel directly from the data of acoustic emission measurements during its diagnostics.

This approach significantly expands the capabilities of technical means of systems for identifying the state of metal structures, in particular, it allows assessing the current state of the equipment and its suitability for further operation without stopping the equipment in real time. It is of interest not only to fix the damage, but also to determine the initial stage of changes in the structure of materials on continuously operating equipment.

## 6. Conclusions

1. The method based on the use of the AE phenomenon for assessing the degree of degradation of mechanical properties and residual life of metal structures under complex dynamic deformation stresses is a fundamental step in determining the technical states of objects. The obtained boundary curves of the safety factor for tension and bending make it possible to determine the working capacity area and the failure area of metal structures.
2. The density of the AE signal during bending was shown to be an order of magnitude higher than the density of the AE signal during tension. When diagnosing a sample with a complex nature of loading (longitudinal tension and transverse bending), this property will make it possible to clearly determine the type of mechanical stress in the specimen.
3. Acoustic measurements of the state of the material under load allow the use of only one type of sensor. This approach greatly simplifies the procedure for assessing the residual life of metal structures in continuous operation, and allows the calculation process to be automated.
4. Determination of the density of AE signals under load may serve as an informative diagnostic parameter. Monitoring of acoustic emission signals makes it possible to diagnostic the processes of degradation of mechanical properties during the operation of equipment.
5. The use of the phenomenon of acoustic emission during deformations of tension and bending makes it possible to predict the onset of critically dangerous states of loss of working capacity of metal structures.
6. The presented technology for determining the residual resource, based on the analysis of the acoustic spectrum, makes it possible to determine the state of the equipment in real time.

## Figures and Tables

**Table 1:** International analogues of St3sp grade steel.

Germany	USA	Japan	France	Belgium	Chine
1.0038 St37-3	A284GrD M1017	SS330 SS400	E24-2 S234JRG2	FE360BFN FED1FF	Q235A Q235B

**Table 2:** Chemical composition of St3sp grade steel in percent.

C	Si	Mn	Ni	S	P	Cr	N	Cu	As	Fe
0.18	0.20	0.51	0.25	0.03	0.02	0.2	0.006	0.23	0.06	97.66

## Data Availability

The data presented in this study are available on request from the corresponding author.
